# GM-CSF Armed Oncolytic Adenovirus Enhances T-Cell Infiltration and Suppresses Local and Distal Tumor Growth

**DOI:** 10.3390/v18010102

**Published:** 2026-01-12

**Authors:** Hua-Wei Xu, Qing-Wen Wang, Min Zhao, Jie Jun, Ri-Gan Shu, Yu-Sen Shi, Xiang-Lei Peng, Jie-Mei Yu, Yan-Peng Zheng, Yuan-Hui Fu, Jin-Sheng He

**Affiliations:** College of Life Sciences and Bioengineering, Beijing Jiaotong University, Beijing 100044, China; 21121608@bjtu.edu.cn (H.-W.X.); 22121628@bjtu.edu.cn (Q.-W.W.); 19121614@bjtu.edu.cn (M.Z.); 22121623@bjtu.edu.cn (J.J.); 21121604@bjtu.edu.cn (R.-G.S.); 22121626@bjtu.edu.cn (Y.-S.S.); xlpeng@bjtu.edu.cn (X.-L.P.); jmyu1@bjtu.edu.cn (J.-M.Y.)

**Keywords:** oncolytic adenoviruses, GM-CSF, tumor-infiltrating lymphocytes, abscopal effect

## Abstract

The limited ability of the immune system to infiltrate solid tumors, attributed to the immunosuppressive tumor microenvironment (TME), remains a significant challenge in cancer therapy oncolytic adenovirus (OAd) that can directly kill tumor cells in addition to inducing both innate and adaptive immune responses. Therefore, the use of OAd to treat tumors is an appealing approach. In this study, we engineered an OAd armed with a human granulocyte–macrophage colony-stimulating factor (GM-CSF), controlled by the E2F promoter, Ad5/3-E2F-d24-GM-CSF (named OAd-Z1). The antitumor activity of OAd was tested in vitro and in vivo. These findings demonstrated that OAd expressed GM-CSF, replicated effectively in tumor cells, inhibited tumor growth, activated the de novo antitumor response, promoted apoptosis and immunogenic cell death in tumor cells, and increased cytokine and chemokine production both in vitro and in vivo. Additionally, OAd demonstrated an abscopal effect and stimulated T lymphocyte infiltration in vivo. Our findings demonstrate that OAd-Z1 represents promising immunotherapeutic candidates for lung cancer, with the potential to enhance systemic antitumor immunity.

## 1. Introduction

Lung cancer remains the leading cause of cancer-related mortality in males and the second most common cause in females globally, highlighting the urgent need for innovative therapeutic strategies [1]. While the survival rates for most other cancers have improved in recent years, the 5-year survival rate for patients with lung cancer has improved only slightly. This is primarily because lung cancer patients cannot be diagnosed until the disease has progressed to a late stage, where the chances of survival are poor [2]. Non-small-cell lung cancer (NSCLC) is the most common and deadly type of lung cancer. Although the most common treatment approach for early-stage NSCLC is surgery, the relapse and toxicity rates are high [3]. In many cases of late-stage NSCLC, surgery is no longer an option, and the standard treatment involves concurrent chemoradiotherapy followed by immunotherapy, both of which have been shown to improve patient prognosis [4]. Therefore, immunotherapy holds significant promise for improving patient outcomes, particularly in early-stage diagnosis and advanced disease management, necessitating further investigation.

The suppressive tumor microenvironment is a major obstacle that weakens immunotherapy. Through immunogenic cell lysis, oncolytic viruses (OVs) can redirect the adaptive immune system toward the tumor, thereby twisting the suppressive TME, and increasing susceptibility to immunotherapy. Owing to the release of tumor-associated antigens (TAAs), pathogen-associated molecular patterns (PAMPs), and damage-associated molecular patterns (DAMPs), OVs trigger both innate and adaptive immune responses that targets tumor cells. An increasing number of lymphocytes are recruited during this process, enhancing immune infiltration and alleviating the immunosuppressive tumor microenvironment (TME). The systemic tumor-specific immune response generated by local treatment of primary tumors can eventually affect and inhibit distal tumors, and this phenomenon is called the abscopal effect [5].

Abscopal effect and increased T-cell activation can be induced by immune checkpoint inhibitor (ICI) therapy, which has been widely applied in late-stage lung cancer [6]. The potential of OV therapy combined with other immunotherapies, such as ICIs, is attracting increasing attention, suggesting promising prospects. Clinical and preclinical studies of OVs, in combination with ICIs such as pembrolizumab, have shown that OVs are well tolerated and can increase treatment effectiveness for melanoma [7,8,9], mesothelioma [8,10,11], prostate cancer [12], and ovarian cancer [13], which has not been observed with chemotherapy alone [11,13,14]. Furthermore, it is assumed that an immune-infiltrating status, such as a “hot” TME, rich in tumor-infiltrating lymphocytes (TILs), and immune-stimulating cytokine production (such as type I IFN), is associated with better ICI responsiveness [15]. Therefore, the evaluation of TILs, especially CD8^+^ T cells, is gaining attention, as an immunological biomarker of the abscopal effect [16].

GM-CSF is commonly used in OAd therapies to treat cancer [17]. It enhances the function of neutrophils and macrophages [18], promotes the generation of monocytes [17] and granulocytes [19], and regulates the development, maturation, and differentiation of dendritic cells [20]. Moreover, GM-CSF can directly inhibit tumor cell growth and promote anticancer T-cell responses [21,22]. Blockade of GM-CSF impairs the functionality of T cells [23,24]. Several clinical experiments using GM-CSF-expressing OAd are currently underway [25,26].

In most cancer cells with inactivated Rb, the E2F pathway remains constitutively active, whereas its activity is low in most normal cells or healthy tissues [27]. The adenovirus E1A CR2 region competitively binds to the Rb protein, leading to the release of the transcription factor E2F, which is essential for adenoviral replication in non-tumor cells [28]. Deletion of CR2 restricts adenoviral replication to Rb-inactivated tumor cells [29]. Several oncolytic adenoviruses based on this design strategy have entered clinical trials, such as DNX-2401 and ONCOS-102 (Ad5/3-d24-GM-CSF). DNX-2401 carries a CR2 deletion [30,31], while ONCOS-102 additionally incorporates GM-CSF [12,32]. Building on the advantages of both approaches, we engineered our construct by deleting the CR2 region and inserting an E2F promoter, aiming to achieve efficient replication initiation specifically in tumor cells while enhancing tumor specificity and safety. The virus also carries GM-CSF to potentially improve therapeutic efficacy. During OAd replication, abundant free E2F promoters further activate proliferation-related genes, particularly transcription from the E1 region. High-level replication of oncolytic adenoviruses is beneficial for immune activation [28,30,33].

To elucidate OAd’s ability and potential mechanism to promote immune infiltration and abscopal effect, we designed and performed this study. We constructed OAd expressing GM-CSF and regulated by the E2F promoter, named after OAd-Z1. First, we innovatively confirmed that OAd can activate a de novo antitumor response, leading to the activation of PBMCs and cancer cell death in vitro. We then proved that the treatment with OAd can significantly suppress the growth of lung cancer with a notable abscopal effect. The treatment also activated the infiltration of T lymphocytes and upregulated the production of IFN-γ and other cytokines and chemokines, along with inducing the apoptosis and ICD of cancer cells. Our results provided a fundamental mechanistic explanation for oncolytic virotherapy and combination strategies in NSCLC treatment, while also establishing a theoretical foundation for their further clinical application.

## 2. Materials and Methods

### 2.1. Cell Lines

The human squamous lung carcinoma cell line NCI-H226 (RRID: CVCL_1544, hereinafter referred to as “H226”), murine Lewis Lung Carcinoma (LLC, RRID: CVCL_4358, identical to 3LL (RRID: CVCL_5653)), and LLC-EGFP (RRID: CVCL_RA22) were purchased from the Institute of Basic Medical Sciences of the Chinese Academy of Medical Sciences. HEK293 cells (RRID: CVCL_0045, hereinafter referred to as “293”) were previously maintained in our laboratory. H226 cells were maintained in RPMI 1640 medium. 293, LLC and LLC-EGFP cells were maintained in Dulbecco’s modified Eagle’s medium, supplemented with 10% fetal bovine serum, 100 IU/mL penicillin, and 100 μg/mL streptomycin as well as 2 mM L-glutamine in a humidified incubator at 37 °C and 5% CO_2_. All human cell lines were authenticated via STR profiling within the last three years. All experiments were performed with mycoplasma-free cells.

### 2.2. Oncolytic Adenoviruses and GM-CSF Detection

The oncolytic adenovirus, OAd-Z1, was generated via rescue and propagation in 293 cells, followed by purification via cesium chloride (CsCl) density gradient centrifugation. The replication-deficient adenovirus, H14 (Ad5/3 with deletion of the E1 and E3 regions) was kept in our laboratory. Following purification, viral titers were measured via the Qubit^®^ dsDNA HS Assay Kit (Thermo Fisher Scientific, Beijing, China) and the Adeno-X rapid titer method (Clontech, Mountain View, CA, USA) as previously described [34]. GM-CSF expression in 293, H226 and LLC cells was detected by ELISA kit (PG355, Beyotime, Beijing, China) after 24 h of infection.

### 2.3. The Replication Capacity of Oncolytic Adenovirus in 293, H226 and LLC Cells

293 and H226 cells were infected with OAd at a multiplicity of infection (MOI) of 0.1, and the samples were collected 12, 24, 48, 72, and 96 h after virus infection. The Adeno-X rapid titer method was used to confirm the replication capacity of the oncolytic virus. LLC cells were infected with OAd at MOIs of 10, and the collected samples were detected by qPCR.

### 2.4. Crystal Violet Staining

H226 cells were treated with OAd at different MOIs (MOIs = 0, 0.1, 0.5, 1, 5 and 10). After 96 h, cells were fixed and stained with 2% crystal violet solution. Finally, the crystal violet solution was removed, and photos were taken.

### 2.5. Cell Counting Kit-8 (CCK-8) Assay

H226 cells were treated with OAd at different MOIs (MOIs = 0, 0.01, 0.1, 1, 10 and 100). After 48, 72, 96 and 120 h, CCK-8 solution (Beyotime, C0037, Beijing, China) was added, and cells were incubated at 37 °C, and the absorbance was measured at 450 nm with a microplate reader (Varioskan LUX, Thermo Fisher, Massachusetts, MA, USA). The cell survival rate relative to that of the control was calculated as follows: cell survival rate (%) = (A_MOI=0.01, 0.1, 1, 10 and 100_ − A_blank_)/(A_MOI=0_ − A_blank_) × 100%.

### 2.6. Cell Apoptosis in H226 Cells

H226 cells were treated with OAd at MOIs of 100. After 48 h, cells were fixed and stained with Hoechst 33342. Apoptosis was observed by fluorescence microscope (Ts2R-FL, Nikon, Japan).

H226 cells were treated with OAd at MOIs of 10 for 24, 48 and 72 h. Cells were collected and lysed and analyzed by PARP ELISA kit (JL45693, Jianglaibio, Shanghai, China).

### 2.7. In Vitro Co-Culture and Flow Cytometry (FC)

H226 cells were treated with OAd at MOIs of 5 for 24 h before hPBMCs (PC.00199, Eallbio, Beijing, China) were added at an E:T ratio of 10:1, followed by a 24 h incubation before analysis by flow cytometry. After that, the relative cell death ratio was detected using an LDH kit (C0016, Beyotime, Beijing, China), and the surviving H226 cells were observed by microscope and crystal violet staining. Briefly, cells were collected and incubation with anti-CD8 and anti-CD69 antibodies and analyzed by FC (the antibodies and gating strategy are provided in Supplementary Materials).

### 2.8. Cytokine Assays

H226 cells were treated with OAd at MOIs of 100. After 12 h, the levels of cytokines and chemokines (CCL2, CCL3, CCL5, CXCL10) in the cellular supernatant were measured by corresponding ELISA kits (catalogs are provided in the Supplementary Materials).

### 2.9. Animal Studies

The SPF female C57BL/6 mice, aged 6~8 weeks, were purchased from Vital River Laboratory Animal Technology Ltd. (Beijing, China), and maintained under SPF conditions at the Beijing Laboratory Animal Research Center (Beijing, China). LLC cells (1 × 10^6^ cells) were subcutaneously injected into both flanks of C57BL/6 mice to establish bilateral subcutaneous tumor models. The tumor size was measured every 2 d, and the tumor volume (mm^3^) was calculated as (length × width^2^)/2. After the tumor volume reached approximately 50~100 mm^3^, the mice were randomly assigned to 2 groups according to their tumor volume (*n* = 8) and received intratumoral injections of PBS (20 μL/tumor) or OAd-Z1 (1 × 10^8^ IFU/tumor in 20 μL) every 2 d for 5 times. Samples were collected at 12 d. No mouse mortality occurred during the study. The tumor inhibition ratio was calculated from the relative changes in tumor volume by the following formula: tumor inhibition ratio (%) = $1-\frac{T_{0}-1}{C_{0}-1}\times 100\%$, where T_0_ and C_0_ represent the relative changes in the tumor volume of the OAd and PBS groups between day 10 and day 0, respectively.

### 2.10. GM-CSF in Tumor Tissues

Tissue extraction reagent was added to each tumor sample collected from mice. Samples were homogenized and the supernatants were collected and analyzed by GM-CSF ELISA kit (PG355, Beyotime, Beijing, China).

### 2.11. Killing Capacity of TILs

CD4^+^/CD8^+^ TILs were separated from treated and untreated tumor tissues according to the manufacturer’s protocols (Miltenyi, 130-116-480, Bergisch Gladbach, Germany). TILs were cocultured with target cells (LLC-EGFP) at E:T ratios of 0:1, 1:1 and 10:1 for 24 h. Surviving target cells expressing green fluorescence were observed and photographed by fluorescence microscopy (Ts2R-FL, Nikon, Japan). The relative fluorescence unit (RFU) of each well at an excitation wavelength of 479 nm and an emission wavelength of 517 nm was recorded with a microplate reader.

### 2.12. qPCR

Total DNA was extracted (DP304-02, TIANGEN, Beijing, China) and amplified by the fluorescence-quantitative PCR. The cycling conditions were as follows: 95 °C, 15 min; 95 °C, 15 s; 60 °C, 60 s; for 40 cycles. The primer sequence was as follows: viral hexon protein, forward: 5′-GGTGGCCATTACCTTTGACTCTTC-3′, reverse: 5′-CCACCTGTTGGTAGTCCTTGTATTTAGTATCATC-3′.

### 2.13. RT-qPCR

Total RNA was extracted and reversed (19231ES50&11119ES60, YEASEN, Shanghai, CN). The cDNA was amplified by the fluorescence-quantitative PCR. The cycling conditions were as follows: 95 °C, 15 min; 95 °C, 15 s; 60 °C, 60 s; for 40 cycles. The gene relative expression levels were calculated by the 2^−△△CT^ method. The sequences of primers used were as follows: Bax, forward 5′-CGGCGAATTGGAGATGAACTG-3′, reverse 5′-GCAAAGTAGAAGAGGGCAACC-3′; Bcl-2, forward 5′-ACCGTCGTGACTTGGCAGAG-3′, reverse 5′-GGTGTGCAGATGCCGGTTCA-3′; β-actin, forward 5′-CTCCATCCTGGCCTCGCTGT-3′, reverse 5′-GCTGTCACCTTCACCGTTCC-3′.

### 2.14. Hematoxylin and Eosin (H&E) Staining, Immunohistochemistry (IHC) and Immunofluorescence (IF)

Liver tissues were stained with H&E and analyzed under a light microscope. For all staining protocols, the tissue sections were deparaffinized, rehydrated, and washed in 1% PBST. For IHC, the sections were soaked into 3% hydrogen peroxide to block endogenous peroxidases and incubated with the primary antibodies overnight at 4 °C. The sections were then incubated with HRP-linked antibodies, stained with diaminobenzidine substrate, and counterstained with hematoxylin. In quantitative analyses, the number of positive cells per field of view was plotted on the *y*-axis. Data were collected from three randomly selected fields per section, and the mean value was calculated for statistical comparisons. For IF, the slides (8 μm) were stained with primary antibodies overnight at 4 °C, incubated with fluorescently labeled secondary antibody the next day, and then counterstained with DAPI. Images were acquired by fluorescence microscopy and were analyzed by ImageJ (version: 1.54g). In quantitative analysis, the *y*-axis represents either the count of positive cells per field of view or the relative fluorescence area. Three independent fields were quantified per section, and their mean value was calculated for statistical comparisons. The antibodies used are listed in the Supplementary Materials.

### 2.15. Statistical Analyses

Statistical analysis was performed by the SPSS software version 21 (SPSS, Chicago, IL, USA), and the means of multiple groups were compared by one-way analysis of variance with Tukey’s multiple-comparison test. *p* < 0.05 was considered significant. The data were presented as mean ± SD.

## 3. Results

### 3.1. Generation of OAd Expressing GM-CSF

As shown in Figure S1A, OAd armed with GM-CSF, with the deletion of E1A CR2, E1B 19k and E3 6.7K/gb 19k, was under the control of the E2F promoter. Compared with the replication capability of a replication-deficient adenovirus, H14 (Figure S1B) (*p* > 0.05), the replication capability of these viruses was not compromised by the replacement of promoter and the insertion of the GM-CSF. Moreover, GM-CSF expression was confirmed by ELISA (Figure S1C).

### 3.2. OAd-Z1 Exhibited Potent Oncolytic Activity in H226 Cells, Inducing Apoptosis and Eliciting a De Novo Antitumor Immune Response

**Figure 1:**
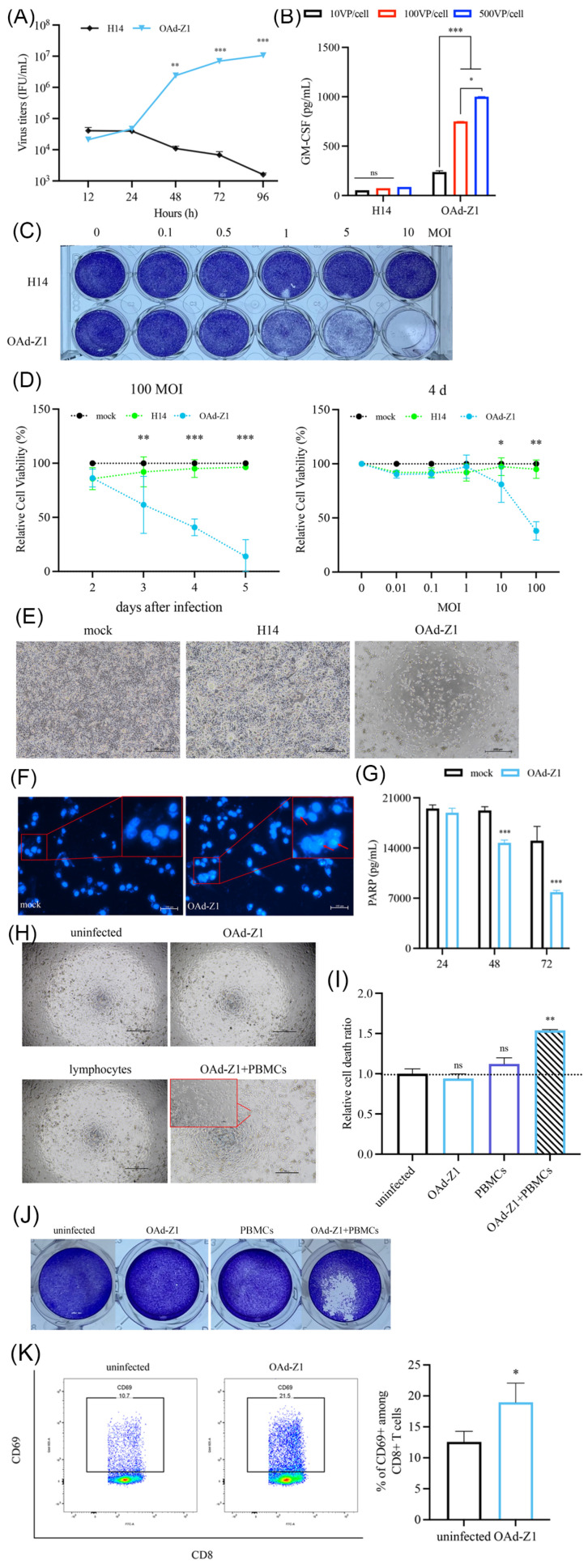
OAd killed H226 cells, upregulated apoptosis and activated de novo antitumor response. (**A**) Viral replication capacity in H226 cells. The figure represents three replicative experiments. (**B**) GM-CSF expression in H226 cells detected by ELISA (*n* = 3). (**C**) Cytocidal effect assessed by crystal violet. (**D**) Cytotoxicity measured by CCK-8 assay (*n* = 3). (**E**) The oncolytic ability was observed by microscope (500 μm). (**F**) Apoptosis induction in H226 cells after 48 h of infection with OAd. Red arrows indicate positive cells. (**G**) PARP expression quantified by ELISA (100 μm, *n* = 3). (**H**) Surviving H226 cells after being cocultured with hPBMCs, observed by microscope (500 μm). (**I**) Relative cell death ratio measured by LDH level in co-culture supernatant (*n* = 3). (**J**) Surviving H226 cells after being cocultured with hPBMCs, observed by crystal violet staining. (**K**) Activated CD8^+^ T cells were measured by CD69 expression (*n* = 3). Data were shown as mean ± SD. (* *p* < 0.05; ** *p* < 0.01; *** *p* < 0.001, ns: not significant.).

As shown in Figure 1A, compared with H14, OAd-Z1 had replication capabilities in H226 cells (*p* < 0.05), indicating that they have intrinsic lytic capabilities in human cancer cells. Similarly, the expression of GM-CSF was detected by ELISA (Figure 1B). Crystal violet staining revealed that OAd-Z1 effectively eradicated H226 cells at an MOI of 10, indicating robust cytocidal activity (Figure 1C). Additionally, as the infection time and MOI increased, the relative cell viability continuously decreased, and more than 70% of H226 cells infected with OAd at 10 MOIs died on day 5 (Figure 1D). This cell-killing effect was similar to the results of crystal violet staining. Furthermore, a cytopathic effect (CPE) was observed in H226 cells (Figure 1E). Similar results were found in A549 cell (Figure S2A–E). These results demonstrated that OAd killed H226 and A549 cells in a dose-dependent and time-dependent manner.

The ability of the deletion of the E1B 19K region to stimulate apoptosis in infected tumor cells has been widely reported and confirmed [35]. Our experiments revealed that OAd-Z1 induced H226 apoptosis, which is consistent with existing studies. Chromosome degradation and compression were detected in the nucleus (Figure 1F) via Hoechst 33342 staining. The ELISA results revealed a significant decrease in the level of PARP (Figure 1G), which is located in the nucleus and is cleaved by activated caspases and is cleaved into cleaved PARP when apoptosis occurs.

OAd can stimulate an antitumor response and activate CD8^+^ T cell response [36]. LDH will be released into the supernatant during cell death; therefore, the relative cell death ratio was calculated as the LDH level compared with that in the uninfected group. The coculture of hPBMCs with OAd-infected H226 cells resulted in cancer cell death, whereas uninfected H226 cells cocultured with hPBMCs remained alive (Figure 1H–J). Interestingly, coculture of hPBMCs with OAd-infected H226 cells significantly upregulated CD69 expression on human CD8^+^ T cells (Figure 1K), suggesting the activation of CD8^+^ T cells. Taken together, these results prove that OAd can evoke specific de novo antitumor effects, stimulate an adaptive immune response and subsequently increase the killing capacity of lymphocytes in the human immune system.

### 3.3. OAd Exhibited Antitumor Efficacy in the LLC Allograft C57BL/6 Mice Tumors Model

**Figure 2:**
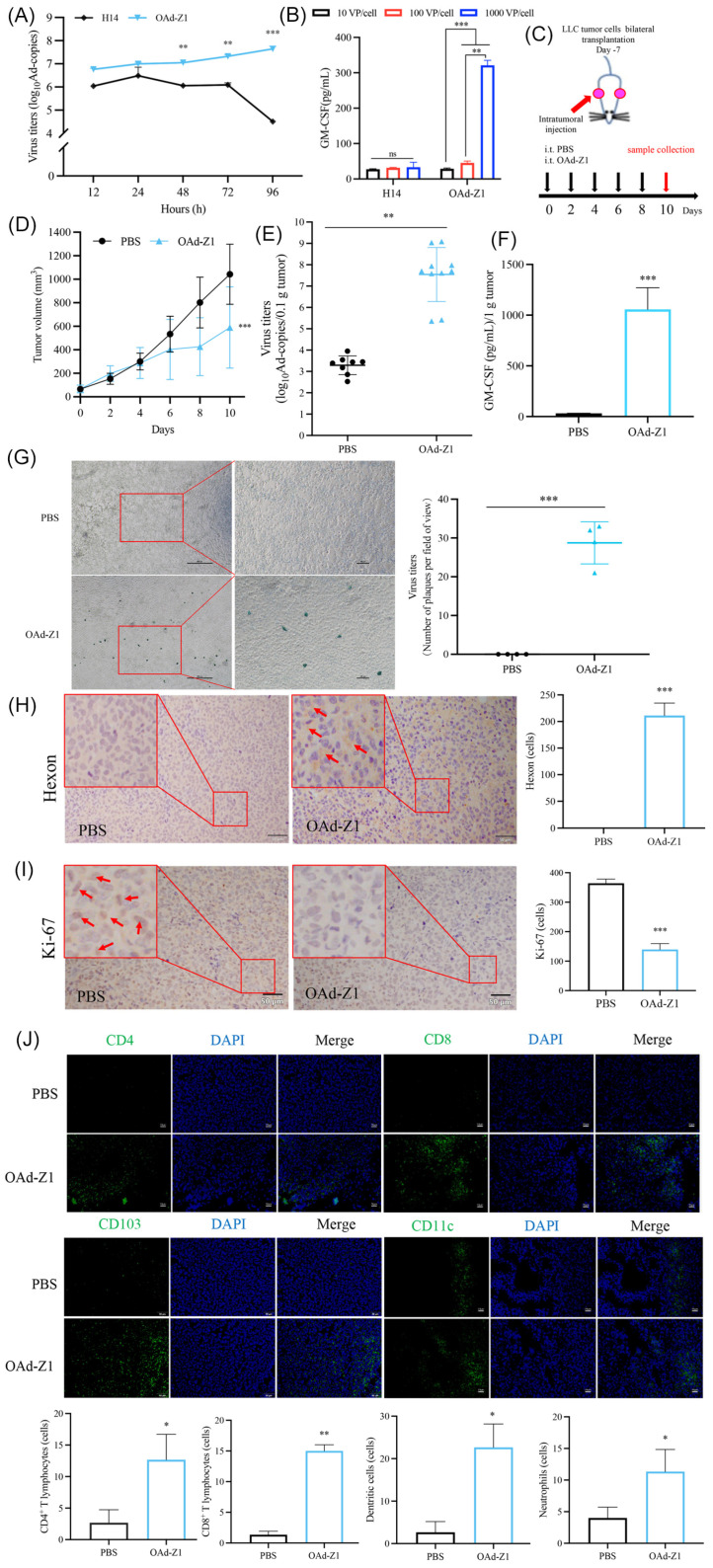
Tumor inhibition capacity and the mechanisms analysis in vivo. (**A**) Virus replication in LLC cells, detected by qPCR (*n* = 3). (**B**) The expression of GM-CSF in LLC cells, detected by ELISA (*n* = 3). (**C**) Therapeutic scheme. In brief, one side was intratumorally administered with OAd (1 × 10^8^ IFU/tumor) every 2 d for 5 times in the bilateral subcutaneous tumor model in C57BL/6 mice, and the other side was untreated. (**D**) The volume of LLC tumors treated with PBS or OAd was monitored until day 10. Statistical analysis on day 10 was performed. (**E**) Virus titers in treated tumor, detected by qPCR (*n* = 8). (**F**) Quantification (tested by ELISA, *n* = 8) of GM-CSF expression in treated tumor tissues harvested 10 d after the initiation of treatment. (**G**) Left panel: Infectious adenovirus particles in treated tumor, detected by plaque assay. Right panel: Number of plaques per field of view. Tumor tissues were harvested on day 10, homogenized, and the supernatant obtained after centrifugation was applied to plaque assay. (**H**,**I**) Representative figures for related antigens expression in treated tumor harvested 10 d after the initiation of treatment were presented by IHC ((**H**) hexon protein, (**I**) Ki-67, 50 μm) and quantification analysis (*n* = 3). Red arrows indicate positive cells. (**J**) Representative figures for CD4^+^ T cells, CD8^+^ T cells, dendritic cells and neutrophils in treated tumor tissues harvested from C57BL/6 10 d after the initiation of treatment were presented by IF and quantification analysis (50 μm, *n* = 3). *p* values comparisons with buffer group were shown. Data were shown as mean ± SD. (* *p* < 0.05; ** *p* < 0.01; *** *p* < 0.001).

Our preliminary study demonstrated that, while lacking statistical significance, OAd-Z1 could inhibit the growth of A549 xenograft in BALB/c nude mice, with the tumor inhibition ratio was 33% (Figure S2F,G), and replicated and expressed within A549 tumors (Figure S2H,I). These results suggested that the oncolytic and cytolytic activities of the OAd alone, along with only innate immune system involvement, might be insufficient for effective tumor suppression. Consequently, we proceeded to investigate tumor inhibition in immunocompetent mouse model. Although the replication ability of adenoviruses was limited in murine cells, the infection and replication (very weak but still present) of OAd, and the expression of GM-CSF were confirmed in LLC cells (Figure 2A,B). Based on the tumor inhibition performance in vitro, we investigated the inhibition of tumor growth by OAd-Z1 in the LLC allograft C57BL/6 mouse tumor model (Figure 2C). Local treatment with OAd significantly inhibited tumor growth (Figure 2D), and the tumor volume of the OAd groups significantly decreased compared with that of the PBS group (*p* < 0.05), where the tumor inhibition ratio was 44%. Ki-67 is a marker antigen of the cell cycle, and it is widely used to assess cell proliferation. We detected the infection, expression and replication of OAd in tumor tissue, as confirmed by the virus titers detected by qPCR (Figure 2E) and plaque assay (Figure 2G), and the expression of GM-CSF (Figure 2F) and hexon protein (Figure 2H). OAd significantly repressed the expression of Ki-67 in tumor tissue, indicating tumor inhibition (Figure 2I). These results indicate that OAd can infect and replicate in LLC tumor cells and inhibit tumor growth.

### 3.4. Treatment with OAd Promoted Immune Cell Infiltration in LLC Allograft Tumors In Vivo

Induction of immune cell infiltration is an important mechanism by which OAd inhibit tumor growth. Therefore, we evaluated the immune infiltration status of tumor tissues. Compared with PBS treatment, OAd-Z1 treatment increased the number of CD8^+^ and CD4^+^ T cells in tumor tissues (Figure 2J). On the basis of the biological function of GM-CSF and its local expression in tumors (Figure 2F), we also focused on dendritic cells and detected their increase (marked by CD103 [37]), which are known as the most powerful antigen-presenting cells (APCs) and are exceptional in initiating de novo immune responses. We also investigated neutrophils, which play an important role in the antitumor effect, and found that the number of neutrophils increased (marked by CD11c). These results indicate that the tumor inhibition effect of OAd treatment may benefit from increased TILs.

### 3.5. Intratumoral Injection of OAd Led to Infection of Uninjected Tumors and Abscopal Effect

**Figure 3:**
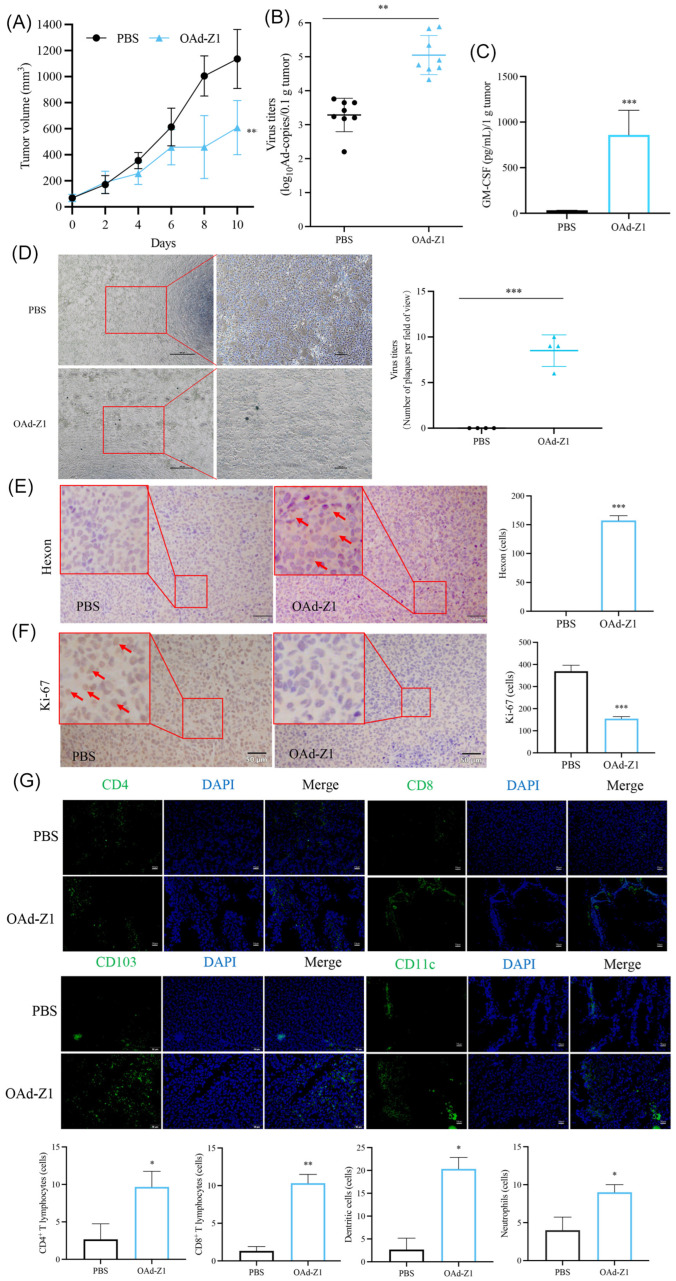
Tumor inhibition capacity and abscopal effect of OAd therapy in LLC allograft tumors in vivo. (**A**) The volume of untreated site of LLC tumors was monitored until day 10. Statistical analysis on day 10 was performed. (**B**) Virus titers in untreated tumor, detected by qPCR (*n* = 8). (**C**) Quantification (tested by ELISA) of GM-CSF expression in untreated tumor tissues harvested 10 d after the initiation of treatment (*n* = 8). (**D**) (**Left**) panel: Infectious adenovirus particles in untreated tumor, detected by plaque assay. (**Right**) panel: Number of plaques per field of view. Tumor tissues were harvested on day 10, homogenized, and the supernatant obtained after centrifugation was applied to plaque assay. (**E**,**F**) Representative figures for related antigens expression in untreated tumor harvested 10 d after the initiation of treatment were presented by IHC ((**E**) hexon protein, (**F**) Ki-67, 50 μm) and quantification analysis (*n* = 3). Red arrows indicate positive cells. (**G**) Representative figures for CD4^+^ T cells, CD8^+^ T cells, dendritic cells and neutrophils in untreated tumor tissues harvested from C57BL/6 10 d after the initiation of treatment were presented by IF and quantification analysis (50 μm, *n* = 3). Data were shown as mean ± SD. (* *p* < 0.05; ** *p* < 0.01; *** *p* < 0.001.).

While the underlying mechanism is still vague, the abscopal effect is an interesting and attractive phenomenon. To evaluate the capacity of OAd-Z1 to induce abscopal effects, we performed another similar experiment but focused on the untreated site. We found that intratumoral injection of OAd also had a significant therapeutic effect at the untreated site (Figure 3A), which is the so-called abscopal effect, where the tumor inhibition ratio was 35%. We also assessed whether the virus spread and infiltrated TILs in the untreated tumors. qPCR, plaque assay, ELISA and IHC were performed, and viral hexon protein and GM-CSF expression were also found in untreated tumors, suggesting that intratumoral injection of OAd led to systemic viral spread (Figure 3B–E). Ki-67 in the tumor cells of the OAd group was much lower than that in the PBS group (Figure 3F). Similarly, we detected a similar immune infiltration status in distant tumors, with increased numbers of CD4^+^ and CD8^+^ T cells, DCs and neutrophils (Figure 3G). In brief, the abscopal effect is the result of both antitumor immunity and systemic viral spread.

To exclude potential interferences from innate immune responses triggered by viral leakage into circulation during intratumoral injection, we also applied a replication-deficient adenovirus control (H14). As shown in Figure S3C,D, a minimal number of infectious viral particles, likely injected virus from the initial administration rather than replicated progeny, along with a low viral copy number, were detected on the treated site. In contrast, neither infectious particles nor viral copies were detected on the untreated site. H14 treatment failed to inhibit tumor growth (Figure S3B), and it further strengthened the conclusion that the observed abscopal effect is mediated by the activation of adaptive immunity. These satisfactory results suggest that the tumor inhibition and abscopal effects of OAd treatment may benefit from increased TILs and show the great potential of OAd in inducing antitumor effects in the whole body, which might improve the outcomes of immunotherapies and promise positive prospects for systemic administration in future exploration.

### 3.6. Killing Capacity of TILs

**Figure 4:**
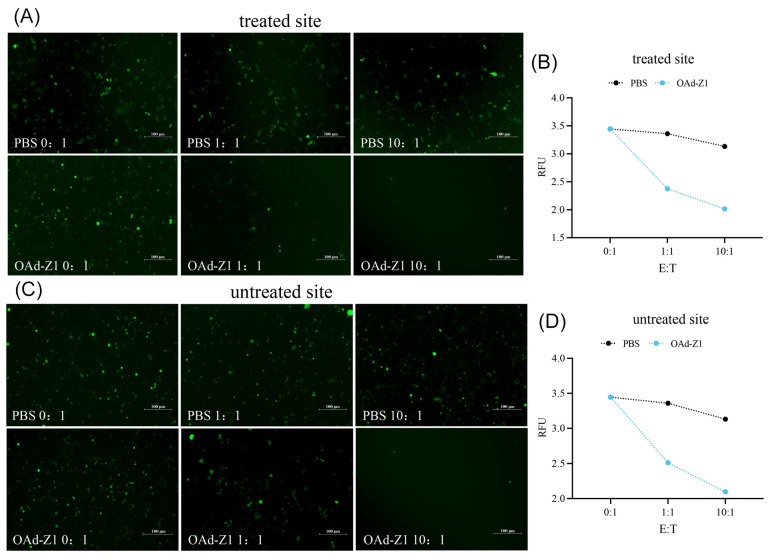
Killing capacity of TILs. TILs were separated from treated or untreated tumor and cocultured with target cells at E:T ratio of 0:1, 1:1 and 10:1, and the surviving target cells were confirmed. (**A**) Representative figures for treated site, observed by fluorescence microscope (100 μm). (**B**) RFU in treated site, recorded by microplate reader. (**C**) Representative figures for untreated site, observed by fluorescence microscope (100 μm). (**D**) RFU in untreated site, recorded by microplate reader. RFU was measured at 479 nm excitation wavelength and 517 nm emission wavelength.

Among all the lymphocytes mentioned earlier, TILs play the most important roles in tumor inhibition and systemic antitumor effects. Therefore, to evaluate the killing capacity of TILs, we separated TILs from both treated and untreated tumors, cocultured them with target cells at different E:T ratios and confirmed the viability of the target cells. After being cocultured with TILs separated from the OAd groups, the number of surviving target cells (expressing green fluorescence) was much lower than that in the PBS group (Figure 4A,C). Furthermore, decreasing the RFU resulted in fewer surviving target cells (Figure 4B,D). These results confirmed the killing capacity of TILs, which may be the main contributor to tumor inhibition.

### 3.7. OAd Induced Immunogenic Cell Death and Apoptosis In Vivo

OAd have been reported to improve the immunogenicity of tumor cells in recent studies [38]. Therefore, tumor cells are more vulnerable to immunotherapies and are much easier to recognize by the immune system. HMGB1 normally exits the nucleus but is released during ICD, and calreticulin (CRT) is known to be an immunogenic molecule associated with immune exclusion. They are identified as “DAMPs” by the immune system and stimulate immune responses to the tumor. Here, we demonstrated enhanced ICD following OAd treatment at both treated and untreated tumor sites (Figure S4A,B), which was a positive pulse to subsequent activated immune infiltration. Immunofluorescence and RT–qPCR confirmed that OAd induced apoptosis in both treated and untreated tumor tissues, as indicated by the accumulation of cleaved PARP (Figure S4B) and Bax mRNA (Figure S4D) and the decrease in the levels of PARP (Figure S4C) and Bcl-2 mRNA (Figure S4D) in vivo.

### 3.8. Treatment with OAd Increased Immune-Stimulating Factors Production In Vitro and In Vivo

**Figure 5:**
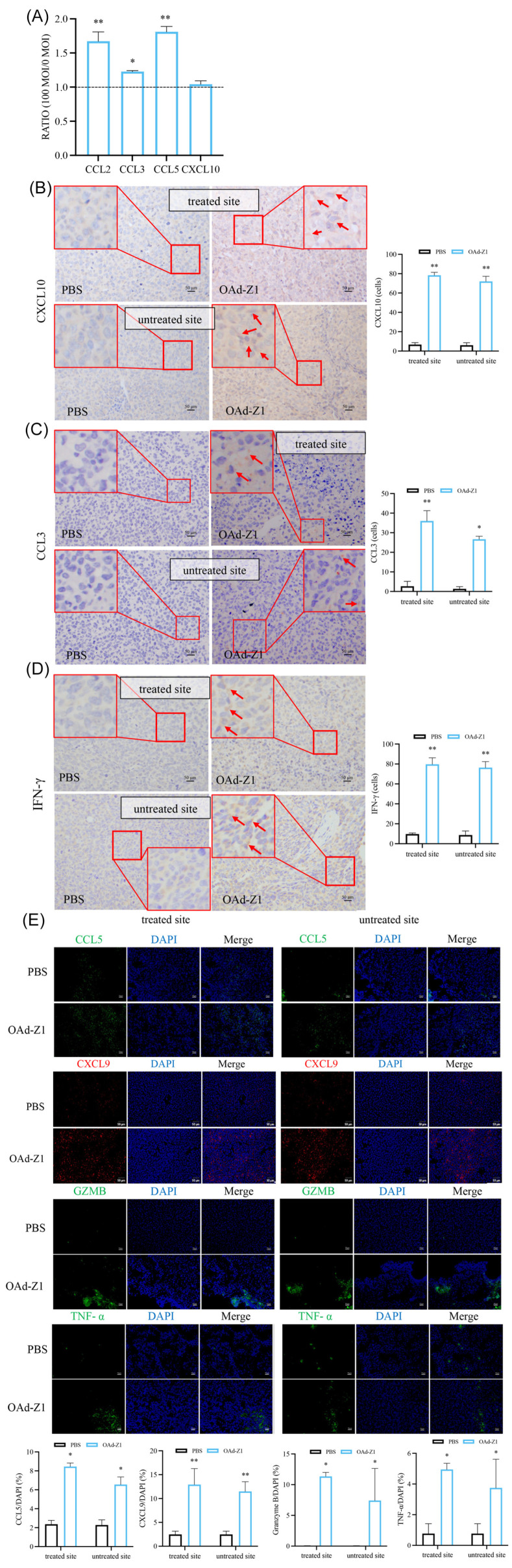
Active release of immunogenic cytokines and chemokines by OAd in vitro and in vivo. (**A**) Cytokines secreted from H226 cells were measured by corresponding ELISA kit, after 12 h of OAd treatment (0 and 100 MOIs), and the ratio of 100 MOIs to 0 MOI was plotted for each cytokine or chemokine (*n* = 3). (**B**–**E**) Representative figures for related antigens expression in treated or untreated tumor tissues harvested from C57BL/6 10 d after the initiation of treatment were presented by IHC ((**B**) CXCL10, (**C**) CCL3, (**D**) IFN-γ) or IF ((**E**) CCL5, CXCL9, Granzyme B, TNF-α, 50 μm) and quantification analysis (*n* = 3). Red arrows indicate positive cells. Data were shown as mean ± SD. (* *p* < 0.05; ** *p* < 0.01).

To analyze the induction of improved immune infiltration, we then evaluated the secretion of proinflammatory cytokines and chemokines. The in vitro release of chemokines such as CCL2, CXCL10 and CCL5, which are known to play important roles in recruiting TILs, was markedly induced (Figure 5A) [39]. These cytokines reflected an acute immune response and a local inflammatory reaction. Moreover, the levels of CXCL10 (Figure 5B), CCL3 (Figure 5C), CCL5 (Figure 5E) and CXCL9 (Figure 5E) in tumor tissues were also found to be increased. They can effectively recruit T lymphocytes or dendritic cells, making the immune-infiltrating status much “hotter”. We also detected increases in Granzyme B (Figure 5E) and IFN-γ (Figure 5D), indicating the presence of cytotoxic T lymphocytes (CTLs). Furthermore, we detected an improvement in TNF-α (Figure 5E), which may enhance the function of TILs and stimulate antitumor effects. Here, we confirmed the increased secretion of proinflammatory factors from cancer cells after treatment with OAd-Z1 both in vitro and in vivo.

### 3.9. Treatment with OAd Stimulated cGAS-STING Signal Pathway In Vivo

**Figure 6:**
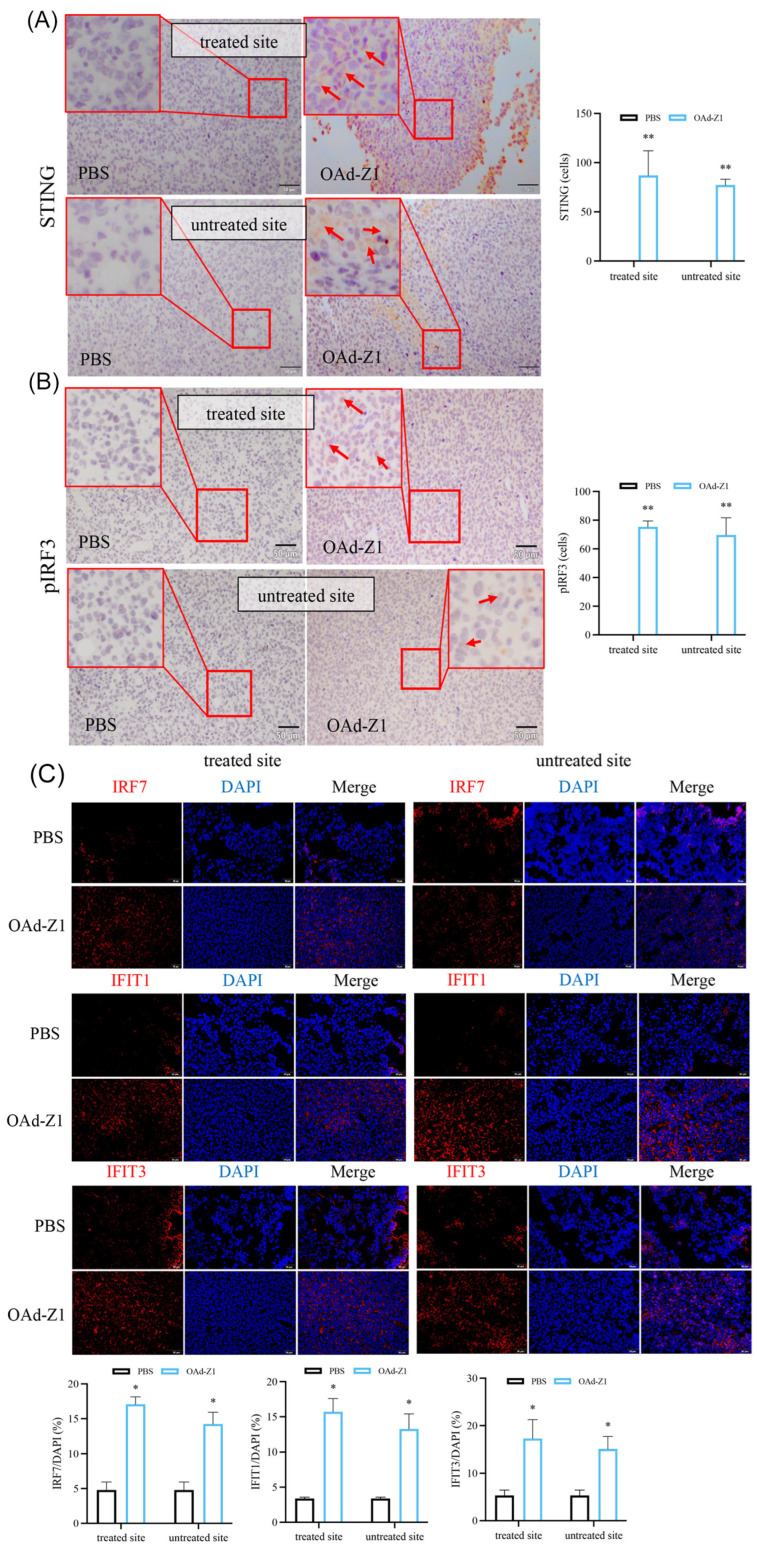
OAd stimulate the cGAS-STING pathway in vivo. (**A**–**C**) Representative figures for related antigens expression in treated or untreated tumor tissues harvested from C57BL/6 10 d after the initiation of treatment were presented by IHC ((**A**) STING, (**B**) pIRF3) or IF ((**C**) IRF7, FIT1 and IFIT3, 50 μm) and quantification analysis (*n* = 3). Red arrows indicate positive cells. Data were shown as mean ± SD. (* *p* < 0.05; ** *p* < 0.01).

The cGAS-STING signaling pathway is stimulated by cytosolic DNA and is related to the production of interferons and apoptosis, leading to intrinsic antitumor immunity, which has drawn much attention [40]. We assumed that adenovirus, as a type of DNA virus, has the potential to stimulate cGAS during infection, transportation, and replication processes. Here, we confirmed the induction of STING, pIRF3, IRF7, IFIT1 and IFIT3 in both treated and untreated tumor tissue after OAd treatment, indicating stimulation of the cGAS-STING pathway (Figure 6). These results may lead to a confirmed explanation for the improved immune infiltration, DC activation, abscopal effects and proinflammatory factor secretion detected.

### 3.10. The Safety of OAd In Vivo

We also assessed the safety of treatment with OAd-Z1. Our results revealed that the weights of the mice in each group increased steadily (Figure S5A), and there was no apparent pathological damage to the liver tissues (Figure S5B). Additionally, there was no detectable hexon protein expression in liver tissues (Figure S5C). The negative results for hexon in total DNA samples extracted from liver, lung and kidney tissues are shown in Figure S5D–F. These results indicate that treatment with OAd at this dose does not cause acute harm or liver trauma.

## 4. Discussion

In this study, we engineered an OAd, OAd-Z1, which exhibited potent tumoricidal activity, including direct tumor cell lysis; inhibition of tumor growth; and induction of de novo antitumor immunity, apoptosis, and immunogenic cell death (ICD), effectively infecting and expressing OAd in tumor tissues. More importantly, they improved CD8^+^ T-cell infiltration and altered cytokine and chemokine secretion patterns, which are essential for lymphocyte recruitment. We also observed an abscopal effect and improved immune infiltration in the untreated site of the tumor.

Indeed, we cannot perfectly analyze the oncolytic ability of OAd in vivo because of the limited replication ability of adenovirus in murine LLC cells and a mouse model. However, since GM-CSF (along with early genes) was successfully expressed in LLC cells, we demonstrated the ability of OAd to trigger immune responses and recruit immune cells, leading to a hotter TME and an abscopal effect. Based on our previous findings in nude mice, where OAd-Z1 only slightly impaired the growth of A549 lung tumors, it is evident that the oncolytic effect of the adenovirus alone, coupled with innate immune engagement, is insufficient for effective tumor suppression. When compared with the results obtained in C57BL/6 mice, it is reasonable to infer that the observed differences, including the abscopal effect, are attributable to the activation of adaptive immunity.

The abscopal effect, a well-documented phenomenon in preclinical models, highlights the systemic antitumor immune response elicited by localized oncolytic virotherapy. Localized treatments with OAd can induce a systemic antitumor effect and reduce the growth of untreated tumors. Jiang et al. reported that treating tumors with oncolytic adenovirus led to the activation, expansion, and migration of in situ T cells to distant untreated tumors [41]. Similarly, Kanaya et al. reported the complete eradication of both treated and untreated tumors via the use of oncolytic adenovirus plus anti-PD-1 [38]. In mice, combined treatment with OAd and ICIs also resulted in improved antitumor responses [42]. The active systemic antitumor immune response after local treatment is now widely considered a significant contributor to the abscopal effect. OAd can immunologically lyse tumor cells and release TAAs, DAMPs, and PAMPs. Additionally, cytosolic DNA, mtDNA, and viral DNA leaked from tumor cells can activate DCs. Native T cells are activated by those DCs and become tumor- or virus-specific CTLs. These CTLs recirculate in the blood and ultimately reach tumors, killing tumor cells and igniting the aforementioned process. Some of these CTLs become long-lived memory T cells [43], which also recirculate in the blood, finally recognizing and killing tumor cells and recruiting more immune cells. Researchers recently demonstrated that after local treatment, OAd encapsulated by tumor-derived extracellular vesicles are generated in local tumor tissue and released [44,45]. These vesicles have tumor specificity, circulate in the blood and finally reach untreated tumors, followed by OAd infection and immune ignition [44]. Here, we can draw a similar conclusion on the basis of our results.

Tumor immunotherapies can be hindered by the inhibitive TME. However, OAd have the potential to reshape the TME and improve tumor immune infiltration. Research has revealed that OAd promote the secretion of cytokines and chemokines, such as CXCL10 and CCL5 [38]. They effectively recruit lymphocytes and restrain Tregs, which ultimately benefits the antitumor immune effect. Studies have also shown that OAd armed with CXCL10 improve the number of CD8^+^ T cells in tumor tissues and enhance their antitumor properties [46]. Similarly, OAd treatment has been reported to increase the expression of CCL5 and M1 characteristics, leading to better efficacy when combined with PD-1 and CAR-T-cell therapies [47].

Our study of OAd-Z1 revealed that they can upregulate the secretion of CCL5 and CXCL10 in tumor cells. This may result in the recruitment of DCs, T cells, NK cells, and other types of cells, ultimately leading to tumor cell death. We also confirmed the promotion of CD4^+^ T cells, neutrophils, GM-CSF, IFN-γ, and TNF-α. CD4^+^ T lymphocytes play a crucial role in controlling tumors by igniting CD8^+^ T lymphocytes and NK cells and releasing IFN-γ, TNF-α, and IL-2 [48]. Neutrophils have anticancer properties through multiple mechanisms, including the induction of antibody-dependent cellular cytotoxicity (ADCC), direct cytotoxic effects, and the activation of adaptive immunity against tumors. GM-CSF, IFN-γ, and TNF-α can induce the differentiation of neutrophils into an antitumor type [49]. Unfortunately, we could not identify CD4^+^ T cells. However, our study revealed that OAd-Z1 can facilitate the recruitment of CD8^+^ T lymphocytes and DCs into tumor tissues. Therefore, OAd-Z1 have the potential to turn immunologically “cold” tumors into “hot” tumors.

By regulating p53-induced apoptosis, OAd promote ICD, which can be normally identified by the extracellular secretion of CRT and high-mobility group box-1 (HMGB1) [50]. Notably, CTLs can also induce tumor cell apoptosis by binding to Fas-L, Fas, and the TNF-α signaling pathway. In addition, it has been widely reported that OVs can cause the apoptosis of tumor cells. A study showed that oncolytic VSV induces apoptosis through the Fas, Daxx, and PKR pathways [51]. Additionally, Ras is redistributed, driven by OVs, resulting in progeny virus release and leading to induced apoptosis [52]. This type of OV-related apoptosis can not only kill tumor cells directly but also activate an antitumor immune effect [53]. Our results are consistent with all these reports.

Although apoptosis induced by OAd indeed leads to the death of tumor cells, they are considered to be immunogenically inert and do not contribute to the highly inflamed microenvironment. However, other types of programmed cell death, such as ferroptosis, necroptosis and pyroptosis, have been reported, drawing increasing attention in antitumor research. CD8^+^ T cells can induce ferroptosis in tumor cells by secreting IFN-γ. Similarly, by secreting granzyme B, CD8^+^ T cells and NK cells can promote pyroptosis, and less than 15% of NK cells in tumor tissue are sufficient to clear an entire tumor graft [54]. Interestingly, our results revealed improvements in the expression of IFN-γ, granzyme B, and HMGB1, suggesting that the types and mechanisms of cell death in tumor tissues are much more complicated.

Cytosolic DNA can bind cGAS and then stimulate the cGAS-STING signaling pathway, which is related to the downstream production of interferons, the activation of NF-kB, and the maturation of DCs. For tumor cells, OAd infect and propagate in them. During the virus replication process, the produced viral DNA or mtDNA leaked from broken mitochondria in tumor cells can activate cGAS, ultimately leading to the upregulated expression of type I IFN [40]. IFNs play a critical role in the antitumor effect. They can induce direct cytotoxic effects on cancer cells and, more importantly, promote the maturation, migration, and antigen presentation of DCs. DCs then initiate a de novo adaptive immune response, therefore linking innate and adaptive immune responses. For DCs, DNA from dying tumor cells or secreted cGAMP in the extracellular environment is taken up and then stimulates the innate cGAS pathway, leading to the expression of interferons and the upregulation of major histocompatibility complex class I (MHCI) and costimulatory molecules such as CD86 [55]. Mature DCs migrate to and activate T cells, which can specifically kill tumor cells. In general, our results demonstrated that treatment with OAd-Z1 stimulated the cGAS-STING signaling pathway, which may enhance the functions of DCs, induce apoptosis and autophagy, and upregulate the expression of proinflammatory factors. These findings may lay a theoretical foundation for the further discovery of combined therapy with OAd and ICIs.

GM-CSF promotes the recruitment and activation of DCs, inducing them to upregulate the expression of OX40L and CD86, followed by increased antigen presentation [56]. GM-CSF significantly enhances tumor growth in an immune-competent Syrian hamster model rather than OAd alone [32]. In five different types of clinical experiments, after being combined with specific cancer vaccines, GM-CSF increased immunocyte counts, such as DC, CD4^+^, and CD8^+^ T-cell counts, and tumor-specific lymphocyte cytotoxicity, which coincides with our results [57]. Recently, the authors suggested that GM-CSF administration approaches combined with ICIs would be beneficial [17]. Here, we used OAd to express GM-CSF in tumor tissues in situ, which resulted in satisfactory tumor inhibition effects and active antitumor responses. Based on these findings, it is reasonable to speculate that the outcomes of OAd-Z1 combined with ICIs would be foreseeable and excellent, which is the next key focus of our subsequent investigations.

However, several limitations should be acknowledged. First, we failed to differentiate between tumor-specific and adenovirus-specific lymphocyte responses. Second, the characterization of the tumor tissue was incomplete. In future investigations, we will incorporate T-cell exhaustion assays, lymphocyte adoptive transfer experiments, and NK cell phenotyping and functional validation to elucidate the underlying antitumor mechanisms involved. It is also important to clarify that our oncolytic adenovirus OAd-Z1 expresses human GM-CSF, which exhibits low biological activity in mice due to the low affinity of the murine GM-CSF receptor α-chain for the human cytokine. This issue could be addressed by using either murine GM-CSF or a humanized mouse model. Nevertheless, based on our current findings, we can reasonably infer that OAd-Z1 would demonstrate stronger tumor-suppressive effects if its full biological activity could be realized.

In summary, we successfully engineered a recombinant oncolytic adenovirus, OAd-Z1. We were satisfied that these viruses effectively killed lung cancer cells and provoked a de novo antitumor response while also promoting apoptosis and ICD. In addition, they increased the expression of chemokines and cytokines and activated immune infiltration in tumor tissues (Figure S6). The abscopal effect and the status of OAd replication and expression in untreated tumor sites suggest that these viruses demonstrate high tumor selectivity and replication and expression capacity. These positive results may benefit from the replacement of the E2F promoter. Our results confirmed that the improved and activated immune infiltration in the tumor could be the reason for the better effect of OAd-immunotherapy strategies. We detected antitumor effects at the untreated site, especially in terms of the tumor inhibition ratio and tumor immune infiltration status, and the production of proinflammatory factors was similar to that at the treated site, which means that these effects might be induced mainly by systemic antitumor immune responses. These findings provide solid evidence for the use of OAd alone or in combination with ICI strategies and future systemic OAd administration for cancer treatment.

## Data Availability

No new data were created or analyzed in this study.

## Supplementary Materials

The following supporting information can be downloaded at https://www.mdpi.com/article/10.3390/v18010102/s1, Figure S1. Construction and characteristic of OAd-Z1.; Figure S2. OAd-Z1 killed A549 cells in vitro, and inhibited tumor growth in A549 xenograft BALB/c nude mice tumor model; Figure S3. Replication-deficient adenovirus cannot inhibit tumor growth; Figure S4. OAd-Z1 induced immunogenic cell death and apoptosis in vivo; Figure S5. The safety of OAd-Z1 treatment in vivo; Figure S6. The mechanisms of the OAd-induced tumor inhibition involve multiple steps.
