# Supplementary material for: GM-CSF Armed Oncolytic Adenovirus Enhances T-Cell Infiltration and Suppresses Local and Distal Tumor Growth
Source: Viruses. 2026 Jan 12;18(1):102. doi: 10.3390/v18010102 (PMC12846385; doi:10.3390/v18010102)
Supplement: Supplementary file 1 [file viruses-18-00102-s001.zip › viruses-4016595-supplementary.pdf]

GM-CSF Armed Oncolytic Adenovirus Enhances T-cell Infiltration and Suppresses Local and Distal Tumor Growth

Supplementary materials

Hua-Wei Xu[Δ1], Qing-Wen Wang[Δ1], Min Zhao[1], Jie Jun[1], Ri-Gan Shu[1], Yu-Sen Shi[1], Xiang-Lei Peng[1], Jie-Mei Yu[1], Yan-Peng Zheng[1*], Yuan-Hui Fu[1*] and Jin-Sheng He[1*]

[1]College of Life Sciences and Bioengineering, Beijing Jiaotong University, Beijing 100044, China

[Δ] These two authors contributed equally.

[*] Corresponding author.
E-mail address: ypzheng@bjtu.edu.cn yhfu@bjtu.edu.cn and jshhe@bjtu.edu.cn

Supplementary figures

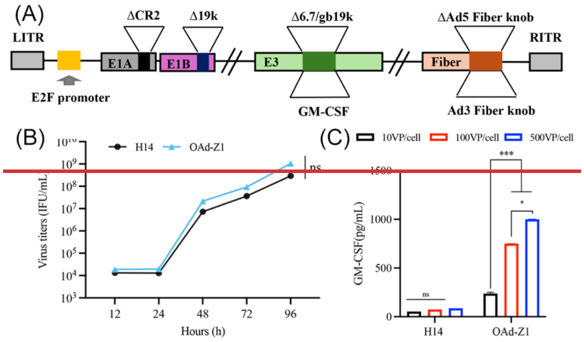

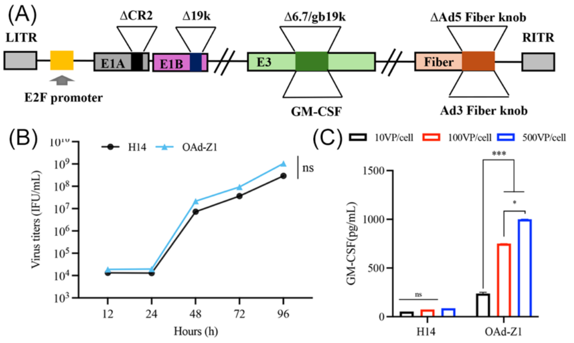

Figure S1. Construction and characteristic of OAd-Z1. (A) Schematic diagram of oncolytic adenovirus construction. OAd arming with GM-CSF, with the deletion of E1A CR2, E1B 19k and E3 6.7K/gb 19k, were under the regulation of E2F promoter. (B) Viral replication capacity in 293 cells was determined by the Adeno-X rapid titer method. The figure represents three replicative experiments. (C) The expression of GM-CSF in 293 cells was detected by ELISA (n = 3). 293 cells were infected with adenovirus at different MOIs, and the supernatants were collected 12 h after virus infection. Data were shown as mean ± SD. (* $P < .05$; ** $P < .01$; *** $P < .001$; ns: not significant.)

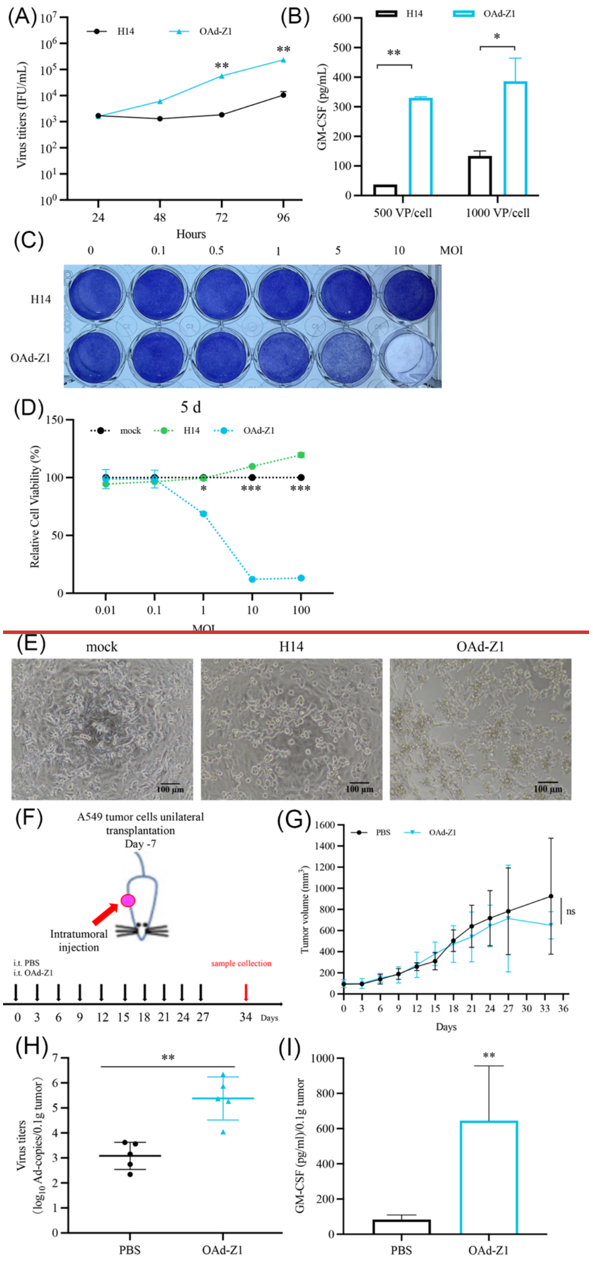

(A)
H14
OAd-Z1
Virus titiers (IFU/mL)
Hours
24
48
72
96
**
**
(B)
H14
OAd-Z1
GM-CSF (pg/mL)
500 VP/cell
1000 VP/cell
**
*
(C)
0
0.1
0.5
1
5
10
MOI
H14
OAd-Z1
(D)
5 d
mock
H14
OAd-Z1
Relative Cell Viability (%)
0.01
0.1
1
10
100
MOI
*
***
***
(E)
mock
H14
OAd-Z1
100 μm
100 μm
100 μm
(F)
A549 tumor cells unilateral transplantation
Day -7
Intratumoral injection
i.t. PBS
i.t. OAd-Z1
sample collection
0 3 6 9 12 15 18 21 24 27 34 Days
(G)
PBS
OAd-Z1
Tumor volume (mm³)
Days
ns
(H)
Virus titers (log10 Ad-copies/0.1g tumor)
**
PBS
OAd-Z1
(I)
GM-CSF (pg/ml)/0.1g tumor
**
PBS
OAd-Z1

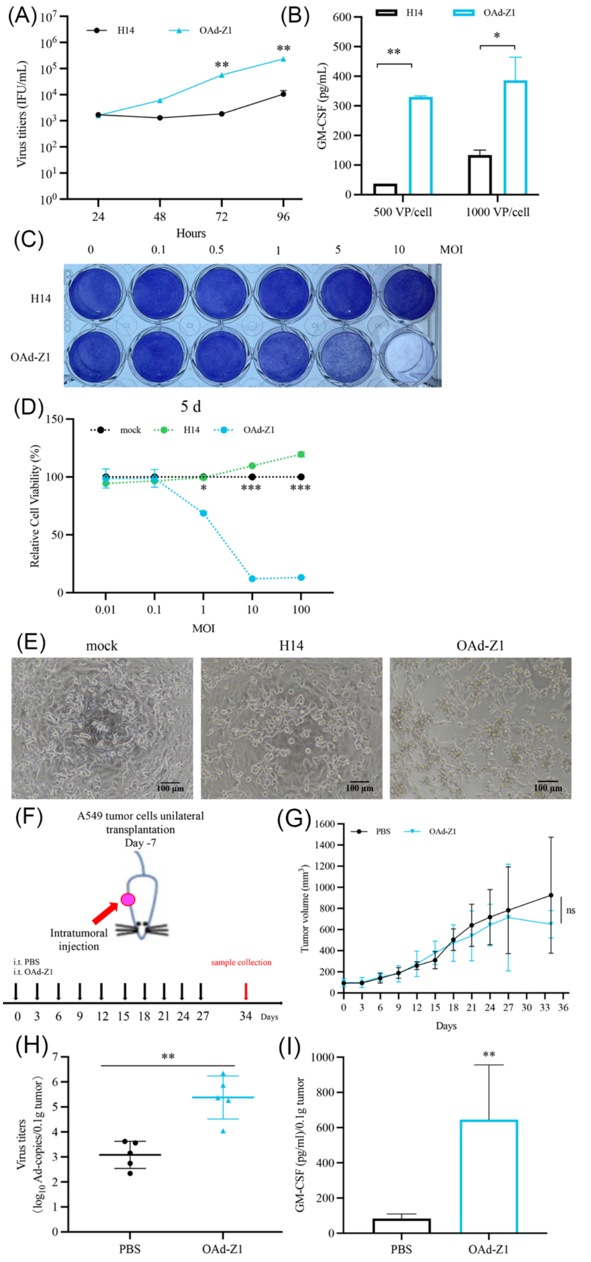

Figure S2. OAd-Z1 killed A549 cells *in vitro*, and inhibited tumor growth in A549 xenograft BALB/c nude mice tumor model. (A) Viral replication capacity in A549 cells. The figure represents three replicative experiments. (B) GM-CSF expression in A549 cells detected by ELISA (n = 3). (C)

Formatted: Not Highlight

Formatted: Font: Not Italic, Not Highlight

Formatted: Not Highlight

Cytocidal effect assessed by crystal violet. (D) Cytotoxicity measured by CCK-8 assay (n = 3). (E) The oncolytic ability was observed by microscope (100 μm). (F) Therapeutic scheme. A549 cells (5×10^6 cells) were subcutaneously injected into one flank of BALB/c nude mice to establish unilateral subcutaneous tumor models. The tumor size was measured every 3 d, and the tumor volume (mm^3) was calculated as (length × width^2)/2. After the tumor volume reached approximately 50~100 mm^3, the mice were randomly assigned to 2 groups according to their tumor volume (n = 5) and received intratumoral injections of PBS (20 μL/tumor) or OAd-Z1 (1×10^8 IFU/tumor in 20 μL) every 3 d for 9 times. Mice were sacrificed 7 days (day 34) after the final administration and the samples were collected. (G) The volume of A549 tumors treated with PBS or OAd-Z1 was monitored until day 34. Statistical analysis on day 34 was performed. (H) Virus titers in tumor, detected by qPCR (n = 5). (I) Quantification (tested by ELISA) of GM-CSF expression in tumor tissues harvested 34 d after the initiation of treatment (n = 5). Data were shown as mean ± SD. (* $P < .05$; ** $P < .01$; *** $P < .001$.)

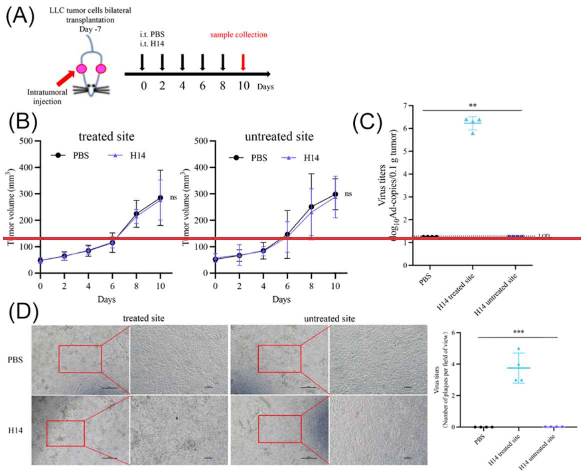

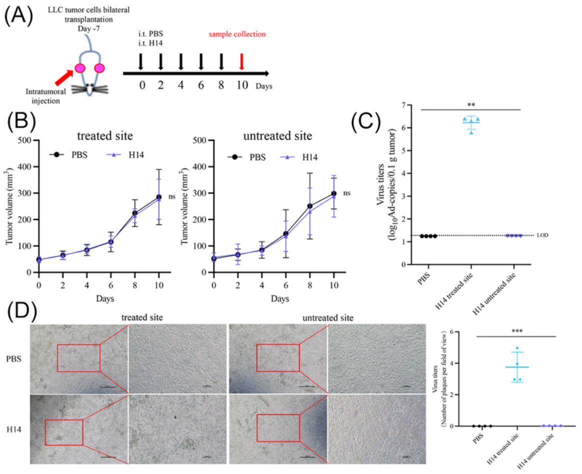

Figure S3. Replication-deficient adenovirus cannot inhibit tumor growth. (A) Therapeutic scheme. LLC cells (1×10^6 cells) were subcutaneously injected into both flanks of C57BL/6 mice to establish bilateral subcutaneous tumor models. The tumor size was measured every 2 d, and the tumor volume (mm^3) was calculated as (length × width^2)/2. After the tumor volume reached approximately 50~100 mm^3, the mice were randomly assigned to 2 groups according to their tumor volume (n = 4) and received intratumoral injections of PBS (20 μL/tumor) or H14 (1×10^8 IFU/tumor in 20 μL) every 2 d for 5 times. Samples were collected at 10 d. (B) The volume of treated or untreated tumors in PBS or H14 group was monitored until day 10. Statistical analysis on day 10 was performed. (C) Virus titers in treated or untreated tumor, detected by qPCR (n = 4). (D) Left panel: Infectious adenovirus particles in treated or untreated tumor, detected by plaque assay. Right panel: Number of plaques per field of view. Tumor tissues were harvested on day 10, homogenized, and the supernatant obtained after centrifugation was applied to plaque assay. Data were shown as mean ± SD. (* P

Formatted: Not Highlight

< .05; ** $P < .01$; *** $P < .001$.)

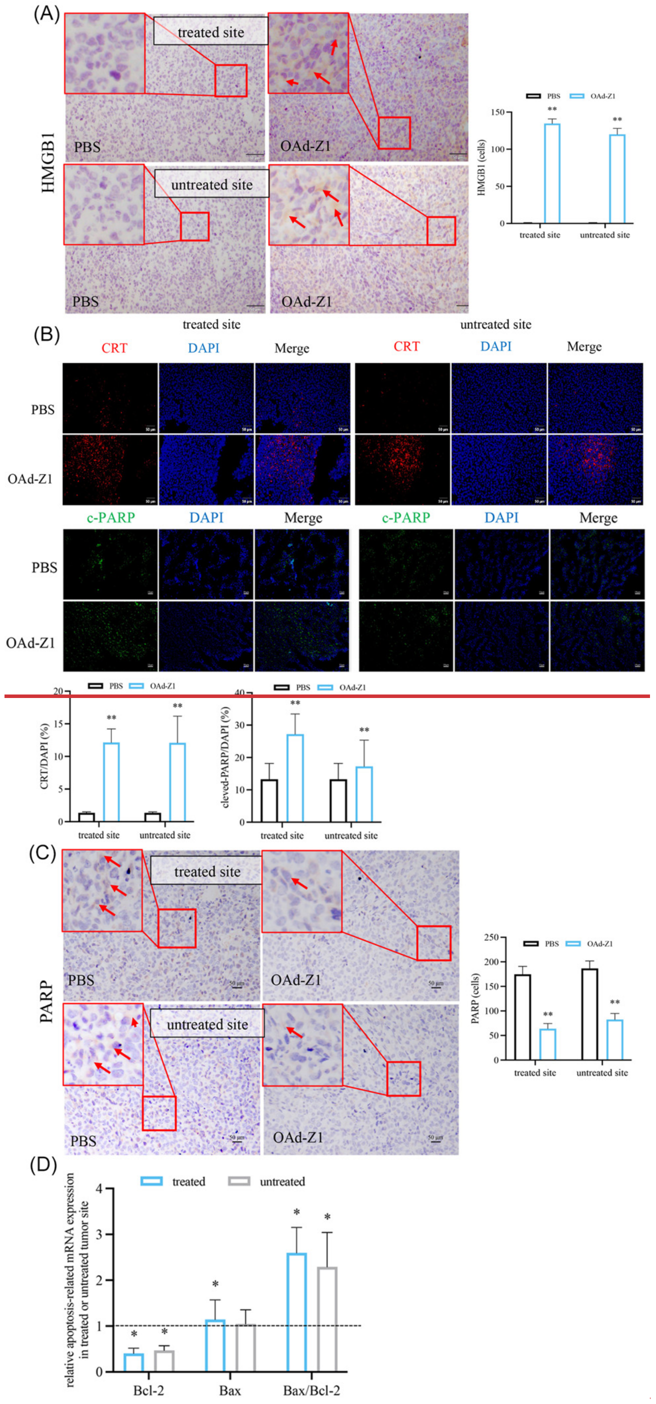
(A)
HMGB1
treated site
untreated site
PBS
OAd-Z1
PBS
OAd-Z1
HMGB1 (cells)
treated site
untreated site
(B)
treated site
untreated site
CRT
DAPI
Merge
PBS
OAd-Z1
c-PARP
DAPI
Merge
CRT/DAPI (%)
cleved-PARP/DAPI (%)
treated site
untreated site
(C)
PARP
treated site
untreated site
PBS
OAd-Z1
PARP (cells)
(D)
treated
untreated
relative apoptosis-related mRNA expression
in treated or untreated tumor site
Bcl-2
Bax
Bax/Bcl-2

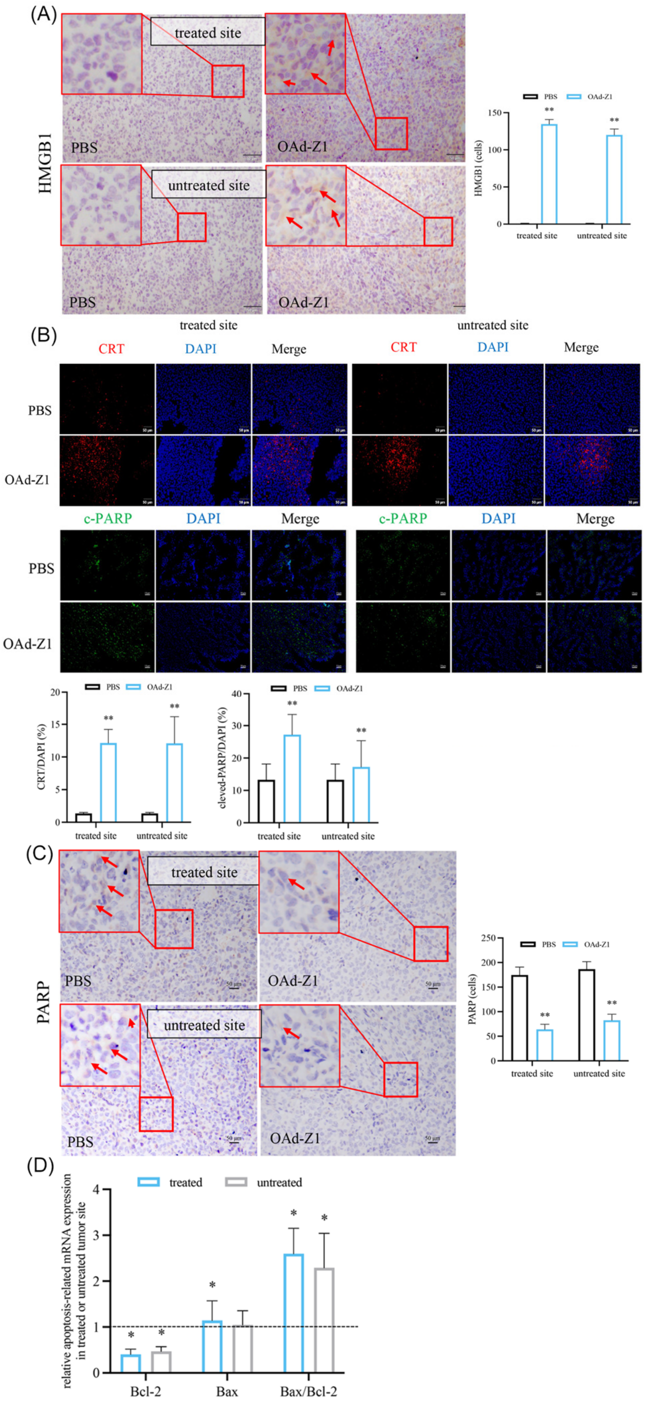
(A)
treated site
HMGB1
PBS
OAd-Z1
untreated site
PBS
OAd-Z1
PBS
OAd-Z1
HMGB1 (cells)
treated site
untreated site
(B)
treated site
untreated site
CRT
DAPI
Merge
PBS
OAd-Z1
c-PARP
DAPI
Merge
PBS
OAd-Z1
PBS
OAd-Z1
CRT/DAPI (%)
treated site
untreated site
cleved-PARP/DAPI (%)
treated site
untreated site
(C)
treated site
PARP
PBS
OAd-Z1
untreated site
PBS
OAd-Z1
PARP (cells)
treated site
untreated site
(D)
treated
untreated
relative apoptosis-related mRNA expression in treated or untreated tumor site
Bcl-2
Bax
Bax/Bcl-2

Figure S4. OAd-Z1 induced immunogenic cell death and apoptosis in vivo. (A) Representative figures for HMGB1 in treated or untreated tumor tissues harvested from C57BL/6 10 d after the initiation of treatment were presented by IHC and quantification analysis (n = 3). Red arrows indicate positive cells. (B) Representative figures for CRT and c-PARP in treated or untreated tumor tissues harvested from C57BL/6 10 d after the initiation of treatment were presented by IF and quantification analysis (n = 3). (C) Representative figures for PARP in treated or untreated tumor tissues harvested from C57BL/6 10 d after the initiation of treatment were presented by IHC and quantification analysis (n = 3). Red arrows indicate positive cells. (D) Relative mRNA expression (tested by RT-qPCR) in treated or untreated tumor tissues harvested from C57BL/6 or 10 d after the initiation of treatment (n = 8). Data were shown as mean ± SD. (* $P < .05$; ** $P < .01$; *** $P < .001$.)

Formatted: Font: Not Italic

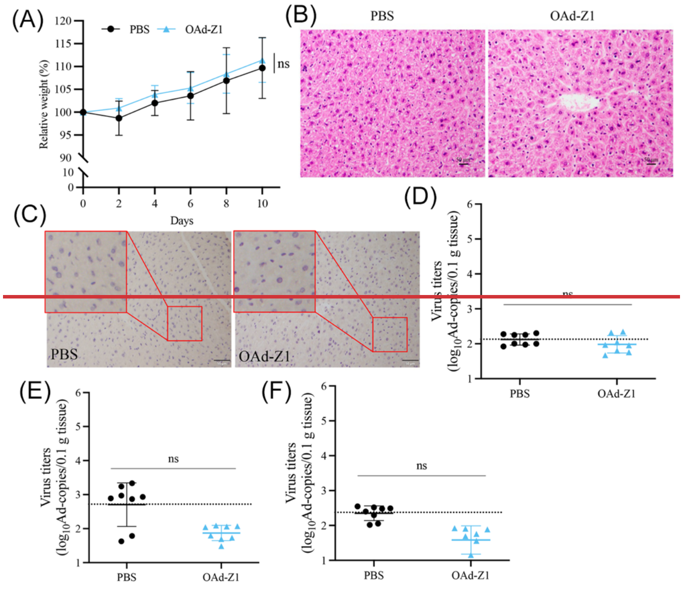

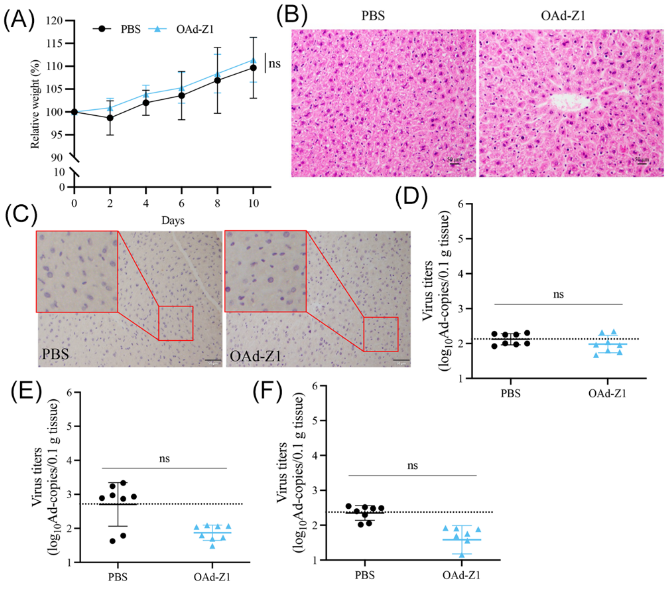

Figure S5. The safety of OAd-Z1 treatment in vivo. (A) The relative weight of C57BL/6 mice treated with PBS or OAd-Z1 was monitored until day 10. Statistical analysis on day 10 was performed (n = 8). (B&C) Representative figures for livers (B) or hexon protein expression in livers harvested from C57BL/6 mice 10 d after the initiation of treatment were presented by H&E staining (B) or IHC (C). (D-F) Virus titers in liver (D), lung (E) and kidney (F), detected by qPCR (n = 8). Data

Formatted: Font: Not Italic

were shown as mean ± SD. ns: not significant.

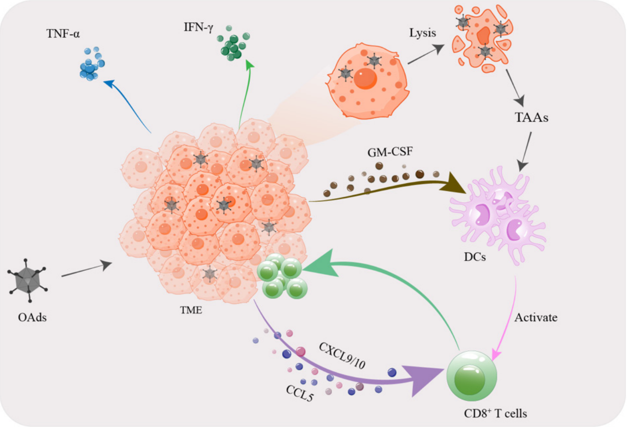

Figure S6. The mechanisms of the OAd-induced tumor inhibition involve multiple steps. OAd infects tumor cells, followed by lysing tumor cells and releasing tumor-associated antigens (TAAs), expressing GM-CSF, and upregulating the expression of immune-stimulating factors. GM-CSF promotes the maturation of dendritic cells (DCs), which will capture TAAs and present to T cells, resulting in the activation of tumor-specific T cells. These chemokines, especially CCL5, CXCL9 and CXCL10, recruit T cells and other immune cells into TME. The cytokines, such as TNF-α and IFN-γ, enhance the functions of immune cells. In summary, OAd treatment makes tumor microenvironment (TME) immunologically active, rendering cancer cells more vulnerable to immunotherapies.

Antibodies

The antibodies used for IHC and IF included anti-Ki-67 (bs-23013R, Bioss, Beijing, CN), anti-Hexon (NB600-1386, Novus, Missouri, MO, USA), anti-CD4 (AF6393, Beyotime, Beijing, CN), anti-CD8 (bs-0648R; Bioss, Beijing, CN), anti-CD103 (BE0026, CST, Massachusetts, MA, USA), anti-CD11c (bs-2508R, Bioss, Beijing, CN), anti-PARP (BD-PT6210, Biodragon, Beijing, CN), anti-cleaved-PARP (BD-PC0101, Biodragon, Beijing, CN), anti-CXCL10 (abs135937, Absin, Shanghai, CN), anti-CCL3 (AF-450-SP, R&D System, Minnesota, MN, USA), anti-IFN-γ (abs135565, Absin, Shanghai, CN), anti-CCL5 (bs-1324R, Bioss, Beijing, CN), anti-CXCL9 (bs-2551R, Bioss, Beijing, CN), anti-TNF-α (AF8208, Beyotime, Beijing, CN), anti-granzyme B (bs-1351R, Bioss, Beijing, CN), anti-HMGB1 (10829-1-AP, Proteintech, Illinois, IL, USA), anti-Calreticulin (27298-1-AP, Bioss, Beijing, CN), anti-STING (19851-1-AP, Proteintech, Illinois, IL, USA), anti-pIRF3 (29528-1-AP, Proteintech, Illinois, IL, USA), anti-IRF7 (22392-1-AP, Proteintech, Illinois, IL, USA), anti-IFIT1 (23247-1-AP, Proteintech, Illinois, IL, USA) and anti-IFIT3 (15201-1-AP, Proteintech, Illinois, IL, USA). The secondary antibodies used in IHC and IF were purchased from ZSGB-Bio (PV-6001, Beijing, CN) and Beyotime (P0193 and P0186, Beijing, CN), respectively.

The antibodies used for FC included FITC anti-human CD8 Antibody (344704, BioLegend, San Diego, CA, USA) and Brilliant Violet 605™ anti-human CD69 Antibody (310938, BioLegend, San Diego, CA, USA).

Gating strategy

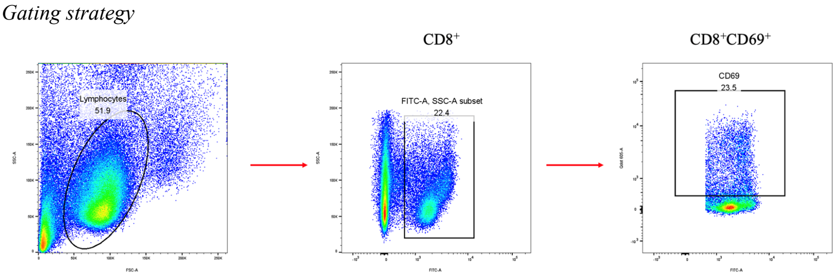

Gates are shown sequentially from left to right.

ELISA and LDH kit

GM-CSF kit (PG355, Beyotime, Beijing, CN), PARP kit (JL45693, Jianglaibio, Shanghai, CN), CCL2 kit (PC130, Beyotime, Beijing, CN), CCL3 kit (PC145, Beyotime, Beijing, CN), CCL5 kit (PC160, Beyotime, CN), CXCL10 kit (PC208, Beyotime, Beijing, CN), LDH kit (C0016, Beyotime, Beijing, CN). All ELISA experiments were proceeded according to the manufacturers' protocols.
